# Social Network Types in Autistic Adults and Its Associations with Mastery, Quality of Life, and Autism Characteristics

**DOI:** 10.1007/s10803-025-06722-1

**Published:** 2025-01-27

**Authors:** Rinske M. van den Heuvel, Jan-Pieter Teunisse, Tulsi A. Radhoe, Wikke J. van der Putten, Carolien Torenvliet, Si Wen, Michel Wensing, Hilde M. Geurts

**Affiliations:** 1 Leo Kannerhuis, Youz (Parnassia Group), Arnhem/Amsterdam, The Netherlands; 2 HAN University of Applied Sciences, Nijmegen, The Netherlands; 3 Department of Psychology, University of Amsterdam, Amsterdam, The Netherlands; 4 Department of General Practice and Health Services Research, University Hospital Heidelberg, Heidelberg, Germany; 5 Present Address: Donders Institute for Brain, Cognition and Behavior, Radboud University, Nijmegen, The Netherlands; 6 Present Address: College of Language Intelligence, Sichuan International Studies University, Chongqing, China

**Keywords:** Autism, Social support, Social network, Heterogeneity, Quality of Life

## Abstract

Research shows heterogeneity in experiences of social contact and social networks in autistic adults. In this study, we aim to identify clusters of social support networks and investigate associations of clusters with mastery, quality of life, and autism characteristics. Autistic adults (*N* = 381; 45.7% female) aged between 30 and 90 years completed questionnaires on social support characteristics, mastery, autism characteristics, and quality of life. A two-step cluster analysis was used to identify clusters based on social support network items. The cluster analysis revealed three clusters: Cluster 1 (*n* = 238) with two or more close persons, sometimes including a romantic partner; Cluster 2 (*n* = 102) with solely a romantic partner as close person; and Cluster 3 (*n* = 41) without any close persons. Level of emotional support was the most important clustering indicator. People in Cluster 3 reported lower quality of life regarding social relationships and mastery, autism characteristics, and other quality of life scales were similar across clusters. Absence or presence of close persons significantly impacts quality of life regarding social relationships in autistic adults, which highlights the importance of addressing (satisfaction with) social support. In order to enhance quality of life, offering social network interventions to increase social support seems especially relevant for autistic people belonging to Cluster 3.

**Supplementary Information:**

The online version contains supplementary material available at 10.1007/s10803-025-06722-1.

## Introduction

In the general population, experiencing social support is positively related to mental health (e.g., Gariépy et al., 2016; Santini et al., 2015). Research in autistic adults on social support and social networks is relatively limited, especially in middle-aged and older autistic adults (Charlton et al., 2022). However, we know that social support is a consistent predictor of quality of life and well-being in autistic individuals (Bailey et al., 2020; Bishop-Fitzpatrick et al., 2018; Charlton et al., 2022; Deserno et al., 2017; Leader et al., 2021; Tobin et al., 2014). In addition, concepts close to social support seem to play a role in mental health in autistic adults. For example, higher levels of social isolation are related to higher levels of social anxiety and depression (Schiltz et al., 2021), and increased loneliness is related to higher levels of psychological distress (Moseley et al., 2021). Also, vice versa, satisfaction with received social support is related to decreased depression symptoms (Gotham et al., 2014). Mental health problems are known to have an elevated prevalence among autistic individuals compared to the general population (Hollocks et al., 2019; Hudson et al., 2019; Lai et al., 2019), with lifetime prevalences estimated between 14.4 to 37% for depression (Hollocks et al., 2019; Hudson et al., 2019) and 42% for anxiety disorders (Hollocks et al., 2019). In this study, we further expand the knowledge on social networks of autistic adults, by exploring the typology of social networks and its associations with quality of life, autism characteristics, and sense of mastery.

Mastery, described as the extent to which individuals feel in control over their lives (Pearlin et al., 1981), is thought to be positively influenced by social support (Thoits, 2011). Also, vice-versa, individuals with a high level of mastery might be more able to form a supportive social network (Thoits, 2011). For example, they might be more likely to choose the social activities that they actually experience as favorable or supportive. Indeed, autistic women stated that another person’s belief in their abilities contributed to the belief in their own abilities (Webster & Garvis, 2017). Research indicates that autistic adults report lower levels of mastery compared to non-autistic adults (van Heijst et al., 2020). Mastery was also identified as a key variable to distinguish two validated subgroups in autistic adults (Radhoe et al., 2023b). Membership of these subgroups was predictive of the quality of life experienced by autistic adults up to five years in the future (Radhoe et al., 2024). Besides these findings, little research has been done on mastery within autism populations, although there is research on related concepts that indicate feeling in control over one’s life outcomes might be related to quality of life in autistic adults. For example, a study in autistic adults showed that higher levels of psychological empowerment, here operationalized as one’s perception of control, efficacy, and outcome expectations, was related to increased quality of life (White et al., 2018). Also, autism traits seem to affect quality of life indirectly through the mediation of feelings of competence, with the latter conceptualized here as feeling one has skills to make effective choices that lead to achieving expected and valued outcomes (Andrews et al., 2023). Thus, increased understanding of feelings of mastery and its relationship to social support in autistic adults could provide a new entry point to improve quality of life.

Autistic individuals seem more vulnerable in their social support networks compared to non-autistic individuals as they experience lower social support levels (Alvarez‐Fernandez et al., 2017; Bishop-Fitzpatrick et al., 2018), their network is often smaller (van Asselt-Goverts et al., 2015), and they are more socially isolated (Stewart et al., 2024). However, research on this topic also suggests that there is a large heterogeneity in social network aspects within the group of autistic individuals, which is consistent with the notion of large heterogeneity between individuals on the autism spectrum in general (Agelink van Rentergem et al., 2021; Lombardo et al., 2019). For example, social network size and density differed substantially among autistic university students (Lei et al., 2019), with some students having small and dense networks, and others having large networks with low density. Further, social interaction is experienced very differently among autistic adults, where some described this as anxiety-provoking or exhausting, but others as mainly enjoyable (Ee et al., 2019). Struggle to form social connections is frequently reported in autistic students, but the type of reported difficulties ranged rather broadly (Bailey et al., 2020). Some reported a desire for a romantic partner, but apart from that they were relatively satisfied with their friendships, while others felt that forming any meaningful relationship was not possible for them (Bailey et al., 2020). Research on subtypes of social support networks within autistic adults could potentially clarify this heterogeneity in social support experiences.

Studies on individual social network aspects (e.g., network size or specific types of social support such as emotional or practical support) and its associations with external variables such as mastery and quality of life provide us with important information on the role of social networks in the lives of autistic people and on possible targets to improve quality of life. However, adopting a multivariate approach using subtypes instead of individual social network aspects to investigate associations with external variables reduces the complexity of (1) intercorrelation between aspects, and (2) combined effects of multiple aspects on external variables (Radhoe et al., 2021). Studies in other populations, such as individuals with an intellectual disability (Tournier et al., 2021) and individuals with long-lasting mental health conditions such as schizophrenia or bipolar disorder (Sweet et al., 2018), have been able to distinguish typologies of social networks with meaningful associations to well-being. Considering the importance of social support for quality of life in autistic people, interventions aimed at improving social support or social networks in autistic adults have been recommended (Bishop-Fitzpatrick et al., 2018; Moseley et al., 2021).

Knowledge on (1) what network types exist within this group and (2) how specific network types relate to relevant external variables might guide clinicians for which individuals such social network interventions apply most. Also, by relating these network types to feelings of mastery and quality of life, we can better understand the heterogeneous experiences of autistic individuals with social support networks, and better identify what aspects are important for whom. Therefore, the current study explored if we could distinguish different types of social networks in autistic adults, based on both functional (e.g., perceived social support) and structural (e.g., network size) aspects. Additionally, we explored associations between the identified social network types and three external variables: quality of life, autism characteristics, and mastery.

## Methods

### Study Design and Participants

This study used data from a larger, accelerated longitudinal study on Aging & Autism (see for details Geurts et al., 2021). We included all participants that completed the Close Person Questionnaire (CPQ; see Measures for further description) for the first time within the longitudinal study.^1^ Data on mastery, quality of life, and social support were included in previous publications by our group (Radhoe et al., 2023a, 2023b, 2024; van Heijst et al., 2020) and this study provided further analysis of these data. For the longitudinal study on Aging and Autism (Geurts et al., 2021), a "think tank" of older autistic adults provided regular advice on topics such as recruitment, study design and interpretation of results.

Inclusion criteria for participants were (1) no past or present diagnosis of intellectual disability and/or a reported IQ score below 70 in the last five years; (2) sufficient understanding of Dutch language; (3) a reported clinical diagnosis of autism spectrum disorder according to the DSM-5 (American Psychiatric Association, 2013) or clinical diagnosis of autistic disorder, Asperger’s disorder or Pervasive Development Disorder Not Otherwise Specified (PDD-NOS) according to DSM-IV (American Psychiatric Association, 2000); and 4) age between 30 and 90 years. Participants were recruited via mental health institutions, advertisement on client organization websites and newsletters, or social media.

People interested in participating were contacted and received a study information package. After providing informed consent, they filled out the questionnaires online or on paper. See for further details on study design and procedure the published protocol (Geurts et al., 2021). The study was approved by the local ethical review board of the Department of Psychology of the University of Amsterdam (Wave 1 2011-PN-1952 and 2013-PN-2668, Wave 2 2015BC-4270, and Wave 3 2018-BC-9285).

### Measures

#### Close Person Questionnaire (CPQ)

The CPQ is a questionnaire that measures social support using 10 items divided into four subscales (Stansfeld & Marmot, 1992). Emotional Support is measured in four items and the other subscales, Practical Support, Negative Experiences, and Inadequacy of Support, in two items each (Hanssen et al., 2019). Social support items were completed for up to three close persons. That is, first participants rated the social support items for a romantic partner if they had indicated having one *(Do you currently have a spouse or someone you consider your steady partner?*). Then, they rated the same items for up to two other close persons if they had indicated having one (*Among your family or roommates, friends, or acquaintances, is there a person (besides your spouse/partner, if any) with whom you feel very connected and/or who gives you emotional or practical support when you need it?*). For the two close persons other than the romantic partner, the type of relationship (parent; sibling; child; other relative; or a friend who is not a relative) and the frequency of contact (daily; weekly; 2–3 times a month; once a month; or less than once a month) were asked. General network size was measured in a separate item (*With how many family members, friends and close acquaintances do you have regular and important contact? Please count only people 18 years and older and leave out your roommates. Answer categories: 0–1; 2–5; 6–10; 11–15; 16–20;* > *20*). Reliability of the CPQ, including the Dutch version, has been demonstrated to be acceptable with Cronbach’s alpha ranging from 0.52 to 0.85 for the subscales and adequate convergent validity for the Dutch version (Hanssen et al., 2019)*.*

#### Autism Spectrum Quotient (AQ)

The AQ (Baron-Cohen et al., 2001; Hoekstra et al., 2008) measures characteristics indicative for autism in five domains: social interaction, attention switching, communication, imagination, and attention to details. It consists of 50 items answered on a 4-point Likert scale ranging from ‘definitely agree’ to ‘definitely disagree’. Some items are reversed. A higher total score indicates more autism characteristics, with possible scores ranging from 50 to 200. The Dutch version of the AQ has been found reliable with satisfactory internal consistency (Cronbach’s alpha ranging 0.71–0.81) and adequate test–retest reliability, and adequate construct and criterion validity (Hoekstra et al., 2008).

#### World Health Organization Quality of Life-BREF (WHOQOL-BREF)

Quality of life was measured with the Dutch version of the WHOQOL-BREF (The Whoqol Group, 1998), consisting of four domains: Physical Health, Psychological Health, Social Relationships, and Environment. It has 24 items plus two items measuring the overall quality of life and general health, all on a five-point Likert scale. For the Dutch version, construct and content validity are shown to be good, and reliability was also shown to be adequate with Cronbach’s alpha ranging from 0.66 to 0.80 for the different domains (Trompenaars et al., 2005). Higher scores indicate higher levels of quality of life. Domain scores were transformed to a 0–100 scale following the WHOQOL Manual (WHO, 2012).

#### Pearlin Mastery Scale

The Pearlin Mastery Scale (Pearlin et al., 1981) measures the level of mastery in seven items on a five-point Likert scale ranging from ‘strongly disagree’ to ‘strongly agree’. Reliability has been demonstrated to be reasonable with a Cronbach’s alpha of 0.67 in a Dutch sample (Penninx et al., 1997). Some items are reversed. Sum scores are used in the analyses, with higher scores indicating higher levels of mastery.

#### Demographic

For demographic description of the sample and clusters, information on education level was included by asking for the highest educational degree participants had obtained. This answer was categorized using the Dutch Verhage scale (Verhage, 1964), ranging from 1 (i.e., < 6 years of primary education) to 7 (i.e., university degree). In addition, information on age, sex, living situation, paid employment, and age of diagnosis was recorded.

### Data Analysis^2^

For the cluster analysis, the mean scores for each social support subscale were calculated by averaging the scores of all reported close persons (including the romantic partner), ensuring participants could receive equal scores regardless of the number of close persons reported. If a participant had no close persons, a score of 0 was assigned. The cluster analysis was then conducted using the following CPQ variables: the four mean social support subscale scores, network size, frequency of contact with close member 1 (if not present: a score of 0 was used), frequency of contact with close person 2 (if not present: a score of 0 was used), and partner present (yes/no).

To identify clusters of social support networks, a two-step cluster analysis in SPSS (version 29.0) was conducted. This type of cluster analysis can handle categorical and continuous data simultaneously (Shih et al., 2010), does not require a pre-specified number of clusters (i.e., is data-driven; Yu, 2010) and was found to be a relatively robust clustering technique (Gelbard et al., 2007). In the first step (i.e., pre-clustering), this two-step cluster analysis explores all possible combinations within the data. In the second step, the optimal combination of clusters is automatically determined based on the distance measure Log-likelihood and the Bayesian information criterion (BIC).

To validate the results of the cluster solution, we applied three strategies (Wen et al., 2021). First, the silhouette measure of cohesion and separation had to be at or above zero, to ensure some distance between clusters. Second, one-way ANOVAs and Chi-square tests on continuous and categorical cluster indicators, respectively, identified the importance of individual clustering indicators. Post-hoc tests for significant indicators were conducted to reveal between which clusters these indicators differed. Third, for split-half cross-validations, the sample was randomly split in half and the two-step cluster analysis was repeated on each half. Validation was demonstrated if the same number of clusters and the characteristics of indicators per cluster were comparable between the two halves and the full samples. Cohen’s κ indicated the level of agreement of assigning participants to the clusters of the different samples.

To investigate whether autism characteristics, quality of life, and mastery differed between social network clusters, ANOVAs with clustering subtype as between group factor and autism characteristics/mastery as dependent variable and a MANOVA with the subscales of the WHOQoL as dependent variables were performed. For post-hoc tests, a Bonferroni correction was used. These analyses with external variables were conducted using both a frequentist approach in SPSS 29.0.1 and a Bayesian approach in JASP 0.18.1.0 with a default prior (i.e., 0.5). Bayes Factor_10_ (BF_10_) indicates the probability that the alternative hypothesis is true compared to the null hypothesis, while BF_01_ indicates the opposite. A BF_10_ of < 1 means no evidence for the alternative hypothesis (i.e., clusters differ on external variables) over the null hypothesis (i.e., no difference between clusters), 1–3 anecdotal, 3–10 substantial, 10–30 strong, 30–100 very strong, and > 100 extreme evidence for the alternative hypothesis (Wagenmakers et al., 2011). We performed robustness checks to check the stability of the BFs under different prior specifications. As for ANOVAs, a robustness check is not yet a standard option in JASP, we checked this by manually changing the prior to 0.2 and 1.0 (van Doorn et al., 2021).

## Results

### Sample Characteristics

In total, 381 of the 404 participants met the inclusion criteria and were included in the cluster analysis. Reason for exclusion of data analyses were reporting a past or present diagnosis of intellectual disability (*n* = 6), CPQ not completed (*n* = 16), or > 10% of CPQ items missing (*n* = 1)*.* Participants were on average 52 years old. See Table 1 for further descriptive characteristics.

**Table 1:** Descriptive Statistics of Demographic in the Full Sample and Across Clusters, Plus Cluster Comparison

	Full sample (*N* = 381)	Cluster 1 (*n* = 238, 62.5%)	Cluster 2 (*n* = 102, 26.8%)	Cluster 3 (*n* = 41, 10.8%)	*F*(*df*_1_, *df*_2_)/ χ^2^(*df*)	*p*-value
Age *M* (*SD*); range	52.3 (12.5); 30–89	50.6 (11.9); 30–89	56.0 (13.2); 30–81	52.8 (12.4); 31–84	6.86 (2, 374)	.001
Sex						
*N*_male_	206 (54.1%)	112 (47.1%)	75 (73.5%)	19 (47.5%)	20.97 (2)	< .001
*N*_female_	174 (45.7%)	126 (52.9%)	27 (26.5%)	21 (52.5%)		
Edu *M* (*SD*); range	5.9 (0.9); 2–7	5.9 (0.9); 2–7	5.9 (0.9); 4–7	5.9 (0.8); 4–7	0.02 (2, 374)	.977
Paid employment					0.94 (4)	.919
*N*_fulltime_	46 (17.1%)	31 (18.3%)	11 (16.4%)	4 (12.1%)		
*N*_parttime_	71 (26.4%)	43 (25.4%)	19 (28.4%)	9 (27.3%)		
*N*_no_	152 (56.5%)	95 (56.2%)	37 (55.2%)	20 (60.6%)		
Living situation					Fisher’s exact	< .001
Alone independently	165 (43.3%)	120 (50.4%)	13 (12.7%)	32 (78%)		
Partner/children	194 (50.9%)	104 (43.7%)	87 (85.3)	3 (7.3%)		
Roommates	9 (2.4%)	5 (2.1%)	2 (2%)	2 (4.9%)		
With parents	6 (1.6%)	4 (1.7%)	0	2 (4.9%)		
Assisted living	7 (1.8%)	5 (2.1%)	0	2 (4.9%)		
Age of autism diagnosis *M* (*SD*); range	46.1 (13.2); 4–81	44.1 (12.9); 4–81	49.9 (12.8); 15–76	48.4 (14); 8–80	7.64 (2, 372)	< .001

*Edu* education, *Alone* alone independently, *Partner/children* independent with partner and/or children, *Roommates* independent with roommates

### Cluster Analysis

The two-step cluster analysis indicated three clusters, which we examined for reliability using three strategies. First, the silhouette measure of cohesion and separation was 0.3, which indicates a fair distance between clusters. Second, the one-way ANOVAs and Chi-square tests showed that the clusters differed significantly on all cluster variables (see Table 2). Third, the cluster analyses in two random subsamples indicated very similar cluster solutions as in the full sample, both in number of clusters and in predictor importance. There was a very high level of agreement in assignment of participants to the clusters of the full sample and the two subsamples (κ = 0.971 and κ = 0.959).

**Table 2:** Results of Cluster Variables from Explorative Cluster Analysis, Plus Cluster Comparison

Clustering indicators^**1**^	Cluster 1 (*n* = 238, 62.5%)	Cluster 2 (*n* = 102, 26.8%)	Cluster 3 (*n* = 41, 10.8%)	*F*(*df*_1_, *df*_2_)/ χ^2^(*df*)	Cluster 1 vs 2 (*p*)^2^	Cluster 1 vs 3 (*p*)^2^	Cluster 2 vs 3 (*p*)^2^
Emotional support *M* (*SD*); range	14.3 (2.1); 8–54	14.9 (2.6); 8–34	0 (0); 0–0	861.45 (2, 379)*	.042	< .001	< .001
Contact frequency network member 1				Fisher’s exact*			
No network member 1	0 (0)	96 (94.1%)	41 (100%)				
Daily	32 (13.4%)	0 (0%)	0 (0%)				
Weekly	100 (42.0%)	0 (0%)	0 (0%)				
2–3 times a month	48 (20.2%)	2 (2%)	0 (0%)				
Once a month	28 (11.8%)	4 (3.9%)	0 (0%)				
Less than once a month	30 (12.6%)	0 (0%)	0 (0%)				
Practical support* M* (*SD*); range	5.6 (1.7); 2–26	7.0 (2.0); 2–18	0 (0); 0–0	223.11 (2, 379)*	< .001	< .001	< .001
Negative experiences* M* (*SD*); range	4.2 (1.4); 2–20	5.1 (1.8); 2–14	0 (0); 0–0	195.77 (2, 379)*	< .001	< .001	< .001
Inadequacy of support* M* (*SD*); range	4.8 (1.6); 2–24	5.3 (1.9); 2–11	0 (0); 0–0	184.32 (2, 379)*	.017	< .001	< .001
Contact frequency network member 2				Fisher’s exact*			
No network member 2	82 (34.5%)	102 (100%)	41 (100%)				
Daily	20 (8.4%)	0 (0%)	0 (0%)				
Weekly	63 (26.5%)	0 (0%)	0 (0%)				
2–3 times a month	26 (10.9%)	0 (0%)	0 (0%)				
Once a month	26 (10.9%)	0 (0%)	0 (0%)				
Less than once a month	21 (8.8%)	0 (0%)	0 (0%)				
Network size				Fisher’s exact*			
0–1	19 (8%)	30 (29.4%)	14 (34.1%)				
2–5	135 (56.7%)	44 (43.1%)	19 (46.3%)				
6–10	53 (22.3%)	19 (18.6%)	6 (14.6%)				
11–15	15 (6.3%)	5 (4.9%)	1 (2.4%)				
16–20	5 (2.1%)	4 (3.9%)	1 (2.4%)				
More than 20	11 (4.6%)	0 (0%)	0 (0%)				
Relationship status				Fisher’s exact*			
In relationship	126 (52.9%)	102 (100%)	0 (0%)				
No relationship	112 (47.1%)	0 (0%)	41 (100%)				

NB Social support scales are averaged per completed close person in this cluster analysis. * = *p* < .001

^1^Clustering variables are presented in order of importance for the cluster solution

^2^Bonferroni correction applied to *p*-value of post-hoc tests

### Cluster Description

The cluster analysis resulted in a three-cluster solution (see Table 2). Emotional support emerged as the most important clustering indicator. The first cluster consisted of participants with at least two close persons (sometimes including a romantic partner) providing social support. Participants in Cluster 2 had a romantic partner but most of them have no other close person in their network. Both the positive (Emotional and Practical support) and negative (Negative experiences and Inadequacy of support) social support scores were highest for Cluster 2 participants across the three clusters. Participants in Cluster 1 had similar levels of Emotional Support compared to participants of Cluster 2, but Cluster 1 participants reported lower Negative Experience and Practical Support scores than Cluster 2 participants. Participants in Cluster 3 had no close persons (including no romantic partner), resulting in social support scores of 0. In terms of demographic characteristics, Cluster 2 consisted of considerably more males than females (73.5% vs 26.5%, respectively), while the number of males and females was roughly equal in the other two clusters.

### External Variables: Autism Characteristics, Mastery, and Quality of Life

Autism characteristics, mastery, and quality of life scores of the three clusters were compared (see Table 3). The BFs appeared to be very stable (see sTable 3). Considering both frequentist and Bayesian results, the only robust difference between the three clusters was found on the Social Relationship quality of life scale, with extreme evidence for this difference. Post-hoc analysis indicated that Cluster 3, with participants without close persons, scored significantly lower than Cluster 2 (*p* < 0.001; BF_10_ = 54) and lower than Cluster 1 (*p* < 0.001; BF_10_ = 6068.69) on this scale. There was no difference in scores on the Social Relationship scale between Cluster 1, in which participants had at least one close person in addition to some having a romantic partner, and Cluster 2, with participants who had only a romantic partner and no other close person (*p* = 0.481; BF_10_ = 0.37 or BF_01_ = 2.7). There was no evidence for a difference in scores on the other three quality of life scales considering both frequentist and Bayesian statistics. Difference in level of autism characteristics was significant between clusters considering frequentist statistics, with frequentist post-hoc analyses indicating that Cluster 2 scored higher than Cluster 1 (*p* = 0.036), but the BF_10_ indicated only anecdotal evidence for a difference between the three clusters. Mastery did not differ between the three clusters based on both frequentist and Bayesian results.

**Table 3:** Comparison with External Variables across Clusters

	Cluster 1 (*n* = 238, 62.5%)	Cluster 2 (*n* = 102, 26.8%)	Cluster 3 (*n* = 41, 10.8%)					
Variable	*M*(*SD*); range	*M*(*SD*); range	*M*(*SD*); range	*F*(*df*)	*p*	*η2*	BF_10_	BF_01_
AQ	144.6 (18.9); 88–185	150.1 (18.1); 104–187	149.0 (17.0); 98–190	3.59 (2, 377)	.028	.019	1.13	0.89
Mastery	20.2 (5.1); 7–35	19.4 (5.1); 9–31	18.2 (5.2); 10–29	3.01 (2, 376)	.051	.016	0.58	1.71
QoL				3.87^1^ (2, 368)	< .001	.041		
Physical	59.1 (16.6); 21.4–100	59.9 (17.6); 17.9–96.4	51.8 (20.0); 10.7–96.4	3.34 (2, 368)	.037	.018	0.69	1.44
Psychol	51.4 (17.2); 8.3–100	50.8 (17.3); 12.5–95.8	46.8 (16.9); 12.5–79.2	1.19 (2, 368)	.307	.006	0.11	8.83
Social	51.2 (19.4); 0–100	48.0 (18.6); 0–91.7	34.6 (20.7); 0–75	12.03 (2, 368)	< .001	.061	1423.26	< 0.001
Environment	67.1 (15.2); 28.1–96.9	68.3 (15.5); 25–96.9	63.0 (15.1); 37.5–90.6	1.69 (2, 368)	.185	.009	0.17	5.82

NB *BF* bayes factor, *AQ* autismspectrum quotient, *QoL* quality of life, *Physical* physical health, *Psychol* psychological, *Social* social Relationships

^1^Wilks’ Lambda

### Social Support per Network Member

**Fig. 1:**
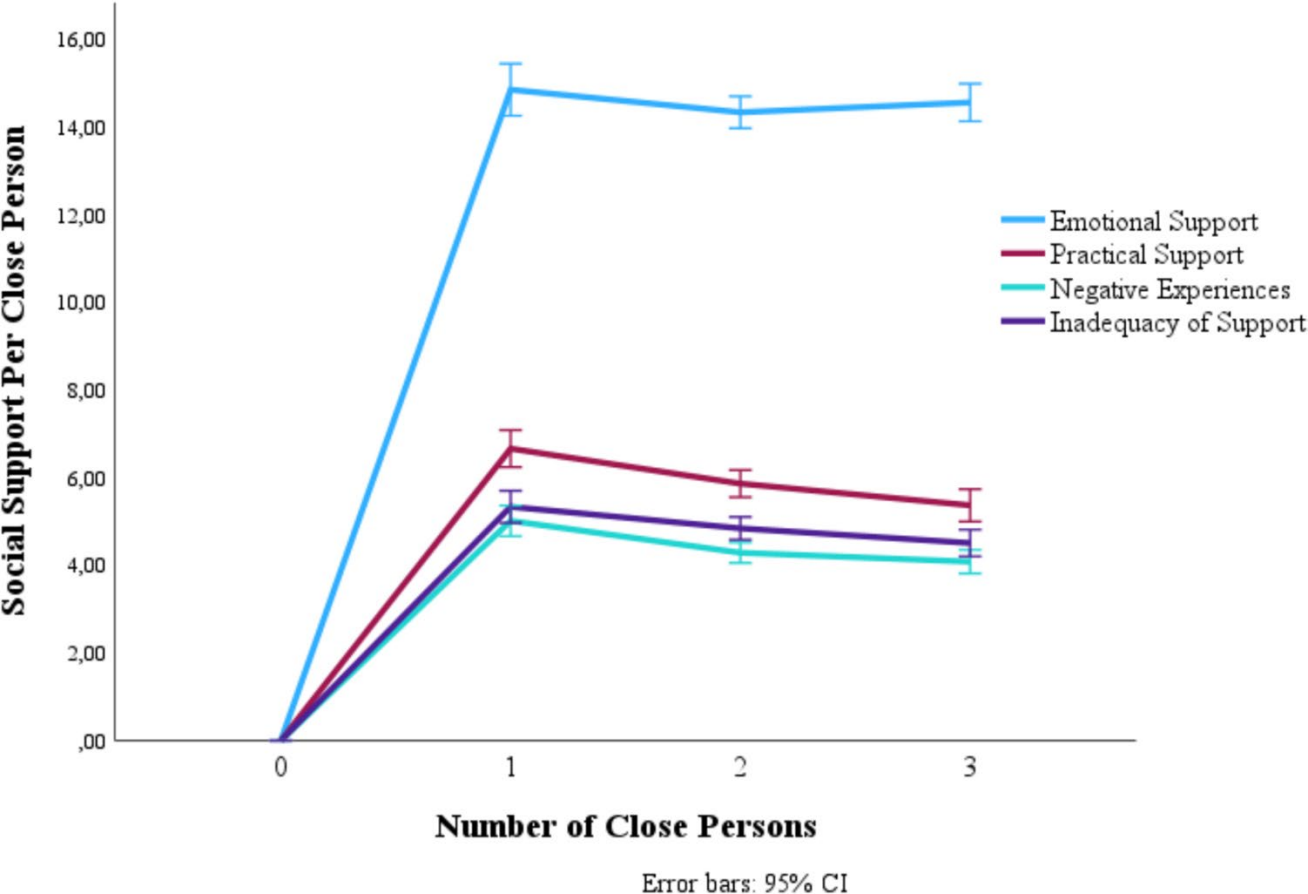
Support per Close Person versus Number of Close Persons

For exploratory purposes, we visualized how the level of social support per close person relates to the number of close persons someone has. As can be observed in Fig. 1, each close person provides a similar amount of support, resulting in an effective increase in the overall amount of social support as the number of network members grows. Thus, this does not indicate that the amount of support each network member provides decreases as the network increases.

Also, we checked what type of relationship was reported for participants' close persons. The most frequently reported type of close person were friends (*n* = 216, 54%), followed by siblings (*n* = 72, 18%), parents (*n* = 61, 15.3%), children (*n* = 40, 10%) or other family members (*n* = 11, 2.8%).

## Discussion

Autistic individuals show large heterogeneity regarding many aspects, including in their social experiences. In this study, we explored whether we could identify meaningful subtypes in social support networks in autistic adults that relate to psychological factors. Three clusters emerged, which can be described as clusters with participants who had: 1) at least two close persons, sometimes including a romantic partner; 2) only a romantic partner as a close person; and 3) no close persons. Here we will summarize and further expand on the results of this study.

First of all, it is important to note that the identified social support network clusters did not differ in autism characteristics or quality of life in terms of physical health, psychological health, and environmental factors. Also, sense of mastery was not related to one of the identified social support network clusters, contrary to what has been theorized before on the relation between mastery and social support (Thoits, 2011). This indicates that clusters of social support networks are not predictive of the external variables examined. This raises the question of whether such associations between social support network types and mastery and quality of life aspects are absent in autistic individuals, or whether they might emerge if other social support characteristics were used in the cluster analysis. As this is a first exploration of social support network clusters in autism, further research is needed to draw firm conclusions about predictive value. Future studies should still consider including mastery as external predictor, given its established links to mental health (e.g., Burns et al., 2011; Dalgard et al., 2007) and quality of life (e.g., Raaijmakers et al., 2014) in other populations, and the limited number of studies that investigated mastery in autistic individuals (e.g., Radhoe et al., 2023b; van Heijst et al., 2020).

Second, it is not unexpected that a small cluster of participants without any close persons emerged (i.e., Cluster 3), given that autistic individuals described both difficulties in establishing and maintaining social relationships, and desires to increase and deepen such contacts (e.g., Ee et al., 2019; Müller et al., 2008; van den Heuvel et al., 2023). A recent study (Stewart et al., 2024) also identified a small group of middle- and older-aged autistic adults who had no family or friends with whom to discuss private matters. The desire for social contact can be ambivalent for autistic people because social situations can be energy demanding and stressful (Kanfiszer et al., 2017; Kock et al., 2019), which can even be enhanced by previous negative social experiences such as being bullied (Ee et al., 2019; Kanfiszer et al., 2017; Sedgewick et al., 2019). The reasons for the absence of close relationships in Cluster 3 are speculative, but we want to emphasize that individuals in this cluster reported lower quality of life about social relationships. So, participants in Cluster 3 seemed to be unhappy with their small social network. This is consistent with research showing that autistic adults experience high levels of loneliness and long for connection and belonging (Grace et al., 2022). Hence, individuals in this Cluster 3 appear particularly crucial to involve in social network interventions to see what they need to satisfy their social needs.

Third, no difference was found in satisfaction with social relationships between those in Cluster 1 (i.e., multiple close persons, sometimes including a partner) and 2 (i.e., only a partner). Apparently, this quality of life domain does not depend on the number of close persons, as long as there is such a close person with whom one has a sense of belonging or support. This is in line with previous findings, for example that satisfaction with one’s network has a more direct effect on well-being than the number of social contacts an autistic individual has (Deserno et al., 2017). Also, a qualitative study found that having one close relationship seemed to work as a buffer for social isolation in autistic older adults (Hickey et al., 2018). Previous research described the feeling of belonging as important for well-being in autistic people, because it makes a person feel understood and accepted (Milton & Sims, 2016). Also, a review of lived experiences of autistic girls and women pointed at the importance of social factors, such as social connectedness, as determinants for well-being and mental health (O’Connor et al., 2024). Inclusivity, support, and acceptance within friendships and relationships were described as important aspects (O’Connor et al., 2024). Feelings of social isolation and loneliness are more common in autistic individuals compared to non-autistic individuals (Grace et al., 2022). This may stem from unmet desires for social contacts (Elmose, 2020), but can also be experienced as a form of isolation within oneself despite having good relationships with others (Hickey et al., 2018). Importantly, lack of autism understanding, and experiences of “othering” as described by autistic people (Elmose, 2020; Milton & Sims, 2016) hinder social connectedness and must continue to be addressed in society to improve quality of life of autistic individuals.

Although participants from the first and second cluster did not differ in their quality of life scores regarding social relationships, one could argue that having only one close person (i.e., such as a romantic partner for those in Cluster 2) can make a person more vulnerable. If circumstances change, for example if that one close person develops problems such as mental or physical health problems, it can become challenging to continue providing social support during this period. Also, some autistic women stayed in negative romantic relationships for fear of not finding another partner easily or because their partner was also their access to their partner’s social circle (Sedgewick et al., 2019). In such situations, it might be more difficult to leave a harmful relationship for autistic adults, which could be concerning if this relationship is the only close person in someone’s network. Also, autistic adults who experienced extreme negative experiences within intimate relationships, such as violence or sexual abuse, reported that having friendships could have served as a protective factor (Douglas & Sedgewick, 2024). Thus, although participants in the second cluster had higher quality of life scores than people in Cluster 3, it may still be in their interest to explore whether their circle of close persons could be broadened.

The current study has some strengths and limitations. A strength of this study is that it focused on middle-aged and older autistic adults, which is an understudied group. Moreover, the use of a multivariate approach contributes to the understanding of social support in autistic adults of this age group. There are also some limitations to keep in mind when interpreting the results of this study. First, the current findings might not be generalizable to all autistic individuals due to characteristics of the sample as 1) participants were often diagnosed with autism later in life (*M* = 46.1; *SD* = 13.2); 2) participants were highly educated and people with a co-occurring intellectual disability were not included; and 3) the sample was not ethnically diverse (i.e., mainly white). However, the participant group might still be representative of many autistic individuals in mental healthcare, as a substantial proportion of participants was recruited from mental healthcare institutions and participants reported high levels of psychological difficulties (see Radhoe et al., 2023b). Still, replication in other, more diverse, autism samples is needed. It would also be insightful to further explore potential gender differences, considering the distinct male/female ratio in Cluster 2 compared to the other clusters, as well as previously documented differences in friendship experiences between autistic girls and boys (Sedgewick et al., 2016). In addition, future research could test temporal stability and predictive validity of the observed clusters (Agelink van Rentergem et al., 2021) to further finetune knowledge on who would benefit most from social network interventions. A second limitation is that support from groups of people, which can be conceptualized as affiliation networks (e.g., Breiger, 1974), were not taken into account. People can feel a connection to a group of people, without having a special or close relationship with one person within this group. For example, attending autism group meetings after a late autism diagnosis offered a sense of shared experience and understanding (Hickey et al., 2018), so social support from group membership seems important to include in future research. Also, previous research showed that autistic individuals viewed professionals as an important source of social support within their social network (van Asselt-Goverts et al., 2015). Thus, in future research, including the option to refer to both one’s affiliation networks as well as professionals as close persons would help to better understand and support the social support needs of autistic adults. A third potential limitation is that the questionnaires used were not designed or tested for autistic individuals specifically, although our autistic think tank was involved in the instructions of each of the measures used. Measures such as the WHO-QOL might miss aspects of quality of life important to autistic individuals (McConachie et al., 2020; Saez-Suanes & Alvarez-Couto, 2021) but so far this has only been tested in the UK setting and it is not clear whether cultural differences impact which additional questions needs to be added. Similarly, the CPQ may not align well with autistic people’s perceptions on social support aspects, and, for example, when a network member is seen as a close person.

Concluding, this study showed that the absence or presence of close persons can have a significant impact on the quality of life on social relationships in autistic adults. This highlights the importance of addressing social support and relationship satisfaction in interventions aimed at improving the quality of life for autistic adults, which has been put forward before (e.g., Moseley et al., 2021).

## Supplementary Information

Below is the link to the electronic supplementary material.

Supplementary file1 (DOCX 33 KB)

## Footnotes

1 Please note that, while we follow our preregistered inclusion criteria, validation strategy, and Bayesian analyses, we pre-registered a slightly different cluster analysis for this study with As Predicted (#105171; https://aspredicted.org/M79_MX6). However, as we made a mistake in the pre-registration of the cluster analysis that would have led to results that were difficult to understand, we chose not to include them here in the main manuscript. Therefore, we decided not to consider this study pre-registered, but to report it as exploratory analyses. For full transparency, we report the results of pre-registered analyses in the supplementary material for those interested.

2 Specifically, we included participants belonging to Wave 1 cohort 1, Wave 2 cohort 2, Wave 3 cohort 1, and Wave 3 cohort 3.
