# Supplementary material for: Social Network Types in Autistic Adults and Its Associations with Mastery, Quality of Life, and Autism Characteristics
Source: J Autism Dev Disord. 2025 Jan 27;56(6):2408–19. doi: 10.1007/s10803-025-06722-1 (PMC13222182; doi:10.1007/s10803-025-06722-1)
Supplement: Supplementary file 1 — Supplementary file1 (DOCX 33 KB) [file 10803_2025_6722_MOESM1_ESM.docx]

**Supplementary Material for “Social Network Types in Autistic Adults and Its Associations with Mastery, Quality of Life, and Autism Characteristics”**

**Table of Content**

1. Results Pre-Registered Cluster Analysis
2. Results Robustness Checks for Bayesian Analyses

**1. Results Pre-Registered Cluster Analysis**

As explained in the footnote of the main manuscript, we made a mistake in the pre-registration (#105171; https://aspredicted.org/M79_MX6) of the cluster analyses and, therefore, decided to report the pre-registered analyses only in the supplementary material. That is, in our pre-registered analyses we planned to use sum scores of the social support subscales of the Close Person Questionnaire (CPQ) as input for the cluster analysis, instead of averaging the scores of all reported close persons. In the CPQ, social support items are only completed for the close persons present. This led to a significant issue when using sum scores: participants with more close persons tended to have disproportionately higher social support scores. As a result, using the sum scores of the CPQ as clustering indicator gave confusing results in the cluster analysis due to difficulty in interpreting the social support levels. In other words, the social support scores captured both perceived social support but also the number of close persons completed. This is problematic because it complicates a clear interpretation of what these social support variables, and therefore the clusters, mean. Therefore, we decided it was better to calculate the social support scores differently so that we could better disentangle this association between level of social support score and number of completed close persons. By using average scores, we could control for differences caused by the number of close persons. For the sake of transparency, we did report the analyses as originally planned in this supplement, but do believe one should be careful when interpreting these findings and we believe these are not as valid as the analyses in the main manuscript.

*Cluster Analysis*

The two-step cluster analysis indicated two clusters (see sTable 1). The silhouette measure of cohesion and separation was 0.4, which indicates a fair distance between clusters. Second, the one-way ANOVAs and Chi-square tests showed that the two clusters differed on all cluster variables, except for relationship status (see sTable 1). Third, the cluster analyses in the two random subsamples indicated very similar cluster solutions as in the full sample, both in number of clusters (i.e., two clusters) and in predictor importance. There was a very high level of agreement in assignment of participants to the clusters of the full sample and the two subsamples (κ = .953 and κ = 1). These results indicated that the cluster solution can be considered as reliable.

*Cluster Description*

The two clusters differed on the level of social support they experience, with Cluster 1 reporting higher levels of emotional and practical social support than Cluster 2 (see sTable 1). Derived from the item on frequency of contact with close network members, Cluster 1 appears to have more close persons than Cluster 2, resulting in more opportunities to experience and report social support. The presence or absence of close network members is also reflected in the item on reported network size. That is, in both clusters, most participants report having 2-5 network members, but a substantial subgroup of Cluster 2 has a network size of 0-1 network members. Clusters do not differ in relationship status. In terms of demographic characteristics, there are relatively more men in Cluster 2 compared to Cluster 1 and the average age is slightly older in the second cluster.

**External Variables: Autism Characteristics, Mastery, and Quality of Life**

Autism characteristics, mastery, and quality of life of the two clusters of the pre-registered cluster analysis were compared (see sTable 2). BFs appeared to be very stable (see sTable 4). Participants in Cluster 2 reported significantly more autism characteristics, with the BF_10_ indicating substantial evidence for this difference. They also had significant lower levels of mastery, but the B_10_ indicated only anecdotal evidence for a difference between the two clusters. The two clusters also differed on the quality of life scales in general. Follow-up ANOVAs showed that this difference was attributable to the Social Relationships scale, where participants of Cluster 1 reported higher quality of life than those in Cluster 2, with BF_10_ indicating extreme evidence for this difference. The Environment and Physical Health scales did not differ between the two clusters and only anecdotal evidence was found for the higher scores in cluster 1 on the Psychological domain.

Supplementary Table 1

*Descriptive Statistics of Clustering Indicators in the Full Sample and Across Clusters, Plus Cluster Comparison.*

|  | Full sample  (*N* = 381) | Cluster 1  (*n* = 214, 56.2%) | Cluster 2  (*n* = 167, 43.8%) | *F*(*df*_1_, *df*_2_)/ χ^2^(*df*) | *p*-value |
| --- | --- | --- | --- | --- | --- |
| Emotional support *M* (*SD*); range | 23.84 (13.68); 0-54 | 33.72 (8.58); 20-54 | 11.19 (6.89); 0-26 | 765.63 (1, 379) | <.001 |
| Inadequacy of support *M* (*SD*); range | 7.93 (4.87); 0-24 | 11.01 (3.80); 4-24 | 3.99 (2.81); 0-10 | 379.91 (1, 379) | <.001 |
| Practical support *M* (*SD*); range | 9.61 (5.79); 0-26 | 13.22 (4.53); 4-26 | 4.99 (3.49); 0-10 | 377.10 (1, 379) | <.001 |
| Contact frequency network member 1 *n* (%) |  |  |  | Fisher’s exact | <.001 |
| No network member 1 | 137 (36) | 0 (0) | 137 (82.0) |  |  |
| Daily | 32 (8.4) | 25 (11.7) | 7 (4.2) |  |  |
| Weekly | 100 (26.2) | 85 (39.7) | 15 (9.0) |  |  |
| 2-3 times a month | 50 (13.1) | 48 (22.4) | 2 (1.2) |  |  |
| Once a month | 32 (8.4) | 27 (12.6) | 5 (3.0) |  |  |
| Less than once a month | 30 (7.9) | 29 (13.6) | 1 (0.6) |  |  |
| Negative experiences *M* (*SD*); range | 7.17 (4.35); 0-20 | 9.85 (3.45); 4-20 | 3.75 (2.66); 0-9 | 355.92 (1, 379) | <.001 |
| Contact frequency network member 2 *n* (%) |  |  |  | 206.14 (5) | <.001 |
| No network member 2 | 225 (59.1) | 58 (27.1) | 167 (100) |  |  |
| Daily | 20 (5.2) | 20 (9.3) | 0 (0) |  |  |
| Weekly | 63 (16.5) | 63 (29.4) | 0 (0) |  |  |
| 2-3 times a month | 26 (6.8) | 26 (12.1) | 0 (0) |  |  |
| Once a month | 26 (6.8) | 26 (12.1) | 0 (0) |  |  |
| Less than once a month | 21 (5.5) | 21 (9.8) | 0 (0) |  |  |
| Network size *n* (%) |  |  |  | Fisher’s exact | <.001 |
| 0-1 | 63 (16.5) | 16 (7.5) | 47 (28.1) |  |  |
| 2-5 | 198 (52) | 116 (54.2) | 82 (49.1) |  |  |
| 6-10 | 78 (20.5) | 50 (23.4) | 28 (16.8) |  |  |
| 11-15 | 21 (5.5) | 15 (7.0) | 6 (3.6) |  |  |
| 16-20 | 10 (2.6) | 7 (3.3) | 3 (1.8) |  |  |
| More than 20 | 11 (2.9) | 10 (4.7) | 1 (0.6) |  |  |
| Relationship status *n* (%) |  |  |  | 0.19 (1) | .67 |
| In relationship | 226 (59.3) | 129 (60.3) | 97 (58.1) |  |  |
| No relationship | 155 (40.7) | 85 (39.7) | 70 (41.9) |  |  |

NB Social support scales are summed for a total of three close persons in this cluster analysis. Clustering variables are presented in order of importance for the cluster solution.

Supplementary Table 2

*Comparison with External Variables across Two Clusters.*

|  | Cluster 1 (*n* = 214, 56.2%) | Cluster 2 (*n* = 167, 43.8%) |  |  |  |  |  |
| --- | --- | --- | --- | --- | --- | --- | --- |
| Variable | *M*(*SD*); range | *M*(*SD*); range | *F*(*df*) | *p* | η*^2^* | BF_10_ | BF_01_ |
| AQ | 144.1 (19.2); 88-186 | 149.8 (17.4); 98-190 | 9.02 (1, 378) | .003 | .023 | 8.46 | 0.12 |
| Mastery | 20.3 (5.1); 7-35 | 19.1 (5.3); 9-31 | 5.35 (1, 377) | .021 | .014 | 1.48 | 0.66 |
| QoL |  |  | 6.39^1^ (1, 369) | <.001 | .065 |  |  |
| Physical | 59.5 (16.4); 21.4-100 | 57.4 (18.5); 10.7-96.4 | 1.3 (1, 369) | .255 | .004 | 0.22 | 4.63 |
| Psychol | 52.5 (16.9); 12.5-100 | 48.5 (17.2); 8.3-95.8 | 5.02 (1, 369) | .026 | .013 | 1.28 | 0.78 |
| Social | 53.0 (18.7); 0-100 | 42.9 (20.0); 0-91.7 | 25.08 (1, 369) | <.001 | .064 | 14675.24 | <0.001 |
| Environment | 68.3 (14.3); 28.1-96.9 | 65.5 (16.5); 25-96.9 | 2.90 (1, 369) | .089 | .008 | 0.47 | 2.15 |

NB BF = Bayes Factor; AQ = Autismspectrum Quotient; QoL = Quality of life; Physical = Physical Health; Psychol = Psychological; Social = Social Relationships

^1^ Wilks’ Lambda

**2. Results Robustness Checks for Bayesian Analyses**

Supplementary Table 3

*Results Robustness Checks for Comparison with External Variables across Three Clusters (Manuscript Version).*

| Variable | BF_10_ with Default Prior (i.e., 0.5) | BF_10_ with More Narrow Prior (i.e., 0.2) | BF_10_ with Wider Prior (i.e., 1) |
| --- | --- | --- | --- |
| Autism Characteristics | 1.13 | 1.13 | 1.13 |
| Mastery | 0.58 | 0.58 | 0.58 |
| QoL Physical health | 0.69 | 0.69 | 0.69 |
| QoL Psychological | 0.11 | 0.11 | 0.11 |
| QoL Social Relationships | 1423.26 | 1423.26 | 1423.26 |
| QoL Environment | 0.17 | 0.17 | 0.17 |

Supplementary Table 4

*Results Robustness Checks for Comparison with External Variables across Two Clusters (Pre-Registered Version).*

| Variable | BF_10_ with Default Prior (i.e., 0.5) | BF_10_ with More Narrow Prior (i.e., 0.2) | BF_10_ with Wider Prior (i.e., 1) |
| --- | --- | --- | --- |
| Autism Characteristics | 8.46 | 8.46 | 8.46 |
| Mastery | 1.48 | 1.48 | 1.48 |
| QoL Physical health | 0.22 | 0.22 | 0.22 |
| QoL Psychological | 1.28 | 1.28 | 1.28 |
| QoL Social Relationships | 14675.24 | 14675.24 | 14675.24 |
| QoL Environment | 0.47 | 0.47 | 0.47 |
